# The ankle sprain and the domino effect

**DOI:** 10.1002/ksa.12538

**Published:** 2024-11-28

**Authors:** M. Dalmau‐Pastor, J. Calder, J. Vega, J. Karlsson, M. T. Hirschmann, G.M.M.J Kerkhoffs

**Affiliations:** ^1^ Human Anatomy and Embryology Unit, Department of Pathology and Experimental Therapeutics University of Barcelona Barcelona Spain; ^2^ MIFAS By GRECMIP (Minimally Invasive Foot and Ankle Society) Merignac France; ^3^ Fortius Clinic London UK; ^4^ iMove Traumatology Tres Torres Barcelona Spain; ^5^ Department of Orthopaedics, Sahlgrenska Academy University of Gothenburg Gothenburg Sweden; ^6^ Department of Orthopedic Surgery and Traumatology Kantonsspital Baselland Bruderholz Switzerland; ^7^ Department of Clinical Research, Research Group Michael T. Hirschmann, Regenerative Medicine & Biomechanics University of Basel Basel Switzerland; ^8^ Department of Orthopedic Surgery and Sports Medicine Amsterdam UMC Location University of Amsterdam Amsterdam The Netherlands; ^9^ Amsterdam Movement Sciences Amsterdam The Netherlands; ^10^ Amsterdam Collaboration for Health & Safety in Sports (ACHSS) International Olympic Committee (IOC) Research Center Amsterdam UMC Amsterdam The Netherlands

It is a pleasure to welcome a new special section of KSSTA dedicated to the ankle joint. This section focuses on medial ankle pathology, and particularly the deltoid ligament, about which there has been a great debate. We hope that this special section will provide a greater understanding of the anatomy and pathology and guide clinicians when they manage medial ankle injuries.

Injuries of the deltoid ligament can be found in isolation, but also as a concomitant lesion with lateral ankle ligament injuries, syndesmosis injuries and/or ankle fractures. Considerable focus has been placed on lateral ankle sprains and a greater knowledge of their anatomy and optimal management has been gained over the past decade. We have moved from considering a lateral ankle sprain as a ‘simple’ injury to realizing that it may lead to a variety of problems if not managed correctly with persistent lateral and medial ankle pain, stiffness, progressive instability and possibly peroneal tendon problems [8, 11, 12, 13, 15, 18].

Refining the anatomy of the lateral ankle ligaments helped to better understand why patients develop chronic symptoms after a lateral ankle sprain [4, 6, 25]; for instance, the anterior talofibular ligament (ATFL) inferior fascicle was shown to be connected to the calcaneofibular ligament (CFL), and the ATFL's superior fascicle was shown to be an intra‐articular structure. The intra‐articular position of an injured ATFL superior fascicle is thought to impair healing, similar to the resynovialization process of a ruptured anterior cruciate ligament remnant in the knee [19]. This theory of impaired healing reinforced the concept of microinstability, originally described in 2016 [26]. Different functions of the ATFL fascicles have also been demonstrated in biomechanical studies [3]. This detailed understanding of the anatomy led to improving the indications for ankle arthroscopic treatments such as arthroscopic lateral ligament repair and reconstruction [22].

The understanding of osteochondral injuries of the talar dome has improved particularly following several publications from the Amsterdam University Medical Center. Their contributions have been key in understanding how the cartilage degradation process in the ankle resembles a cascade [2]. Osteochondral lesions are present in up to 65% of chronic ankle sprains and 75% of ankle fractures [12, 13, 15]. The talar dome is a convex structure but with a concavity in the frontal plane, therefore forming two talar shoulders, one lateral and one medial. During an ankle inversion sprain, there is an impact between the medial talar shoulder and the tibial plafond; this impact can create a microscopic crack in the articular cartilage, invisible at imaging (even micro‐computed tomography), affecting joint biomechanics and initiating the possibility of further joint degeneration [1, 2, 14]. Today, it is well known that not all ankle sprains or fractures lead to ankle joint degeneration and end‐stage ankle osteoarthritis, but it is also known that almost all patients suffering from ankle joint osteoarthritis have a history of ankle trauma. Future research must therefore focus on identifying patients who are at risk of developing ankle osteoarthritis after a history of ankle trauma or repetitive micro‐trauma present in patients with chronic lateral, medial, or syndesmotic (micro)instability or malalignment.

After this short resume, repetition being the mother of all knowledge, it is time to focus on the medial‐sided consequences of inversion ankle sprains, topic of this special section of KSSTA: Koris et al. [16] authored a review on anatomy, diagnosis, and treatment of deltoid ligament pathology that set up the basis of this special section. Reviewing the literature exposed the confusing anatomy of the medial collateral ligament of the ankle joint, the deltoid ligament: as we will see in the anatomy paper published in this section [5], up to 16 fascicles have been described in the deltoid ligament, overcomplicating the description of this ligament. Dalmau‐Pastor et al. present a unifying classification with four different fascicles, highlighting the fact that the deep deltoid (tibiotalar fascicle) and the anterior part of the superficial deltoid (tibionavicular fascicle) are intra‐articular and that their healing capacity may be impaired by a resynovialization process of the ligament after rupture, as happens to other intra‐articular ligaments [19]. The same four fascicles were consistently observed in Fernandez's magnetic resonance imaging study [7], which should facilitate diagnosis.

Regarding treatment, repairing the deltoid ligament is feasible through arthroscopy, even its deep tibiotalar fascicle, as demonstrated in Guelfi et al.'s study [10], and surgical outcomes are promising, either when performed in isolation [23] or together with a lateral ligament repair [9, 17, 24].

Finally, two papers on less frequent yet relevant pathologies are presented: medial malleolus stress fractures [20] and medial sleeve fractures [21]; results of surgical treatment in two groups of elite athletes are presented, highlighting that these pathologies should be included in any differential diagnosis of medial ankle pain.

**Figure 1:**
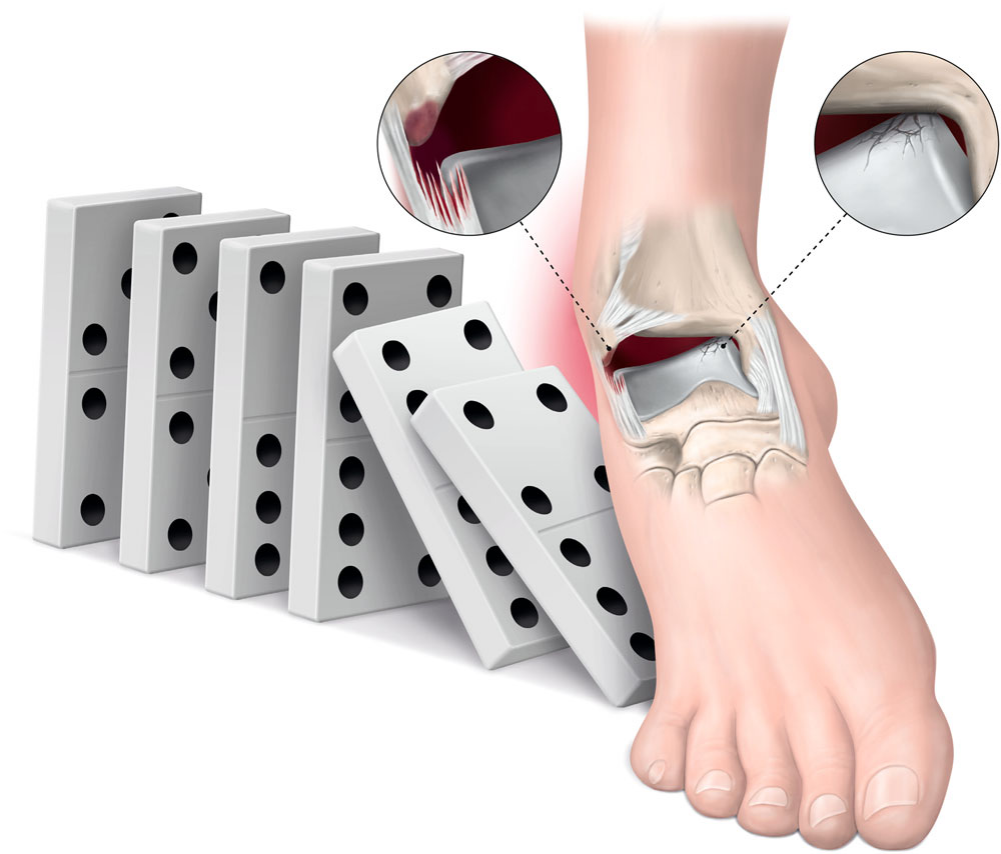
The ankle sprain and the domino effect: An ankle inversion sprain, even in minor cases, can cause damage to ATFL's superior fascicle and to the talar cartilage. That damage might be asymptomatic in the early stage but will still cause altered biomechanics leading to further joint damage, including structures other than the initially injured. ATFL, anterior talofibular ligament.

When all the available evidence is collated, we hypothesize that some anatomical features of the ankle act like domino pieces (Figure 1): ATFL's superior fascicle [6, 25] and the deep deltoid are intra‐articular ligaments [5], likely to have an impaired ability to heal. Despite the ankle being a very congruent joint, the talar dome has two ‘talar shoulders’, which facilitates the impact between talus and tibia seen in inversion sprains, thus enabling the ‘invisible’ crack in the articular cartilage of the talar dome that can then progress into further joint degeneration [1]. It is also known that after a lateral ankle sprain, one or all these structures can be damaged and that in certain patients, each of them will act as a first domino piece, altering joint biomechanics, leading to chronic (micro)instability and so leading further damage to ligaments and cartilage. Hence, we could start to think of a lateral ankle sprain as a first domino piece; if it falls, this leads to further problems in initially non‐injured areas of the ankle joint. One ligament injury may therefore have a global effect on the entire ankle joint. The focus should be to keep all domino pieces standing upright, preventing the domino effect, should one fall. For this, we need more knowledge and hope that this section goes some way into filling some of the gaps in our scientific understanding of ankle injuries.
